# The anthelmintic drug niclosamide induces GSK-β-mediated β-catenin degradation to potentiate gemcitabine activity, reduce immune evasion ability and suppress pancreatic cancer progression

**DOI:** 10.1038/s41419-022-04573-7

**Published:** 2022-02-03

**Authors:** Yangyang Guo, Hengyue Zhu, Yanyi Xiao, Hangcheng Guo, Miaomiao Lin, Ziwei Yuan, Xuejia Yang, Youze Huang, Qiyu Zhang, Yongheng Bai

**Affiliations:** 1 Key Laboratory of Diagnosis and Treatment of Severe Hepato-Pancreatic Diseases of Zhejiang Province, The First Affiliated Hospital of Wenzhou Medical University, Wenzhou, 325000 China; 2 Department of Laboratory Medicine, The First Affiliated Hospital of Wenzhou Medical University, Wenzhou, 325000 China; 3 Human Genetic Resource Bank, The First Affiliated Hospital of Wenzhou Medical University, Wenzhou, 325000 China; 4 Department of Laboratory Medicine, Wenzhou Hospital of Traditional Chinese Medicine, Wenzhou, 325000 China; 5 Department for Hepatopancreatobiliary Surgery, The First Affiliated Hospital of Wenzhou Medical University, Wenzhou, 325000 China; 6 Center for Health Assessment, Wenzhou Medical University, Wenzhou, 325000 China

**Keywords:** Cancer, Cell death, Pharmacology

## Abstract

Niclosamide, a cell-permeable salicylanilide, was approved by the Food and Drug Administration for its anthelmintic efficiency. A growing body of evidence in recent years suggests that niclosamide exhibits potential tumor-suppressive activity. However, the role and molecular mechanism of niclosamide in pancreatic cancer remain unclear. In this study, niclosamide inhibited proliferation of pancreatic cancer cells (PCCs), induced apoptosis via the mitochondrial-mediated pathway, and suppressed cell migration and invasion by antagonizing epithelial-to-mesenchymal transition. Also, niclosamide inhibited tumor growth and metastasis in pancreatic cancer xenograft mouse models. Mechanistically, niclosamide exerted these therapeutic effects via targeting β-catenin. Niclosamide did not reduce β-catenin mRNA expression in PCCs, but significantly downregulated its protein level. Moreover, niclosamide induced β-catenin phosphorylation and protein degradation. Interestingly, niclosamide also induced GSK-3β phosphorylation, which is involved in the ubiquitination degradation of β-catenin. Pharmacological activation of β-catenin by methyl vanillate and β-catenin overexpression abolished the inhibitory effects of niclosamide. Furthermore, niclosamide potentiated the antitumor effect of the chemotherapy drug gemcitabine and reduced the ability of cancer immune evasion by downregulating the expression levels of PD-L1, which is involved in T cell immunity. Thus, our study indicated that niclosamide induces GSK-β-mediated β-catenin degradation to potentiate gemcitabine activity, reduce immune evasion ability, and suppress pancreatic cancer progression. Niclosamide may be a potential therapeutic candidate for pancreatic cancer.

## Introduction

The general view of cancer is that metastasis determines the ultimate outcome of many cancer patients, especially pancreatic cancer patients [1]. Pancreatic ductal adenocarcinoma (PDA) represents approximately 90% of all pancreatic cancers and is one of the top five leading causes of cancer-related deaths [2]. Surgery is regarded as the primary treatment for PDA. However, only a few patients with PDA can undergo surgery. Unresectable locally advanced pancreatic cancer usually invades adjacent important structures, especially the abdominal trunk and superior mesenteric artery. The best treatment method for these patients is controversial. Some patients can try resection after initial treatment. For most patients with locally advanced pancreatic cancer that is absolutely unresectable, or patients with extra-pancreatic lesions that are suspected based on imaging findings but cannot be diagnosed, relevant guidelines recommend chemotherapy for a period of time instead of immediate chemotherapy [3–5]. Gemcitabine is currently one of the main chemotherapeutic drugs used to treat PDA. However, its clinical application fails to prolong the survival time of patients with PDA [6]. Hence, developing novel robust therapeutic strategies to block cancer metastasis or enhance the therapeutic efficacy of gemcitabine is essential to fulfill the unmet needs of patients with PDA.

The role of EMT in PDA has received increased attention across a number of disciplines in recent years. Emerging evidence has shown that the EMT program is a significant driver of PDA progression from initiation to metastasis [7, 8]. Tumor cells undergoing EMT may migrate through the blood stream, threatening manifestations of cancer progression [9]. Many signaling pathways participate in the EMT process of PDA, including Wnt/β-catenin signaling [10, 11]. When Wnt/β-catenin signaling is activated, the nuclear translocation of β-catenin increases, and β-catenin located in the nucleus acts as a coactivator of lymphoid enhancer factors/T-cells to activate downstream genes, such as c-Myc [12]. Wnt/β-catenin signaling also plays an essential role in the proliferation of cancer cells. Abnormal activation of Wnt/β-catenin signaling has been shown in various tumors, including pancreatic cancer [13].

**Fig. 1:**
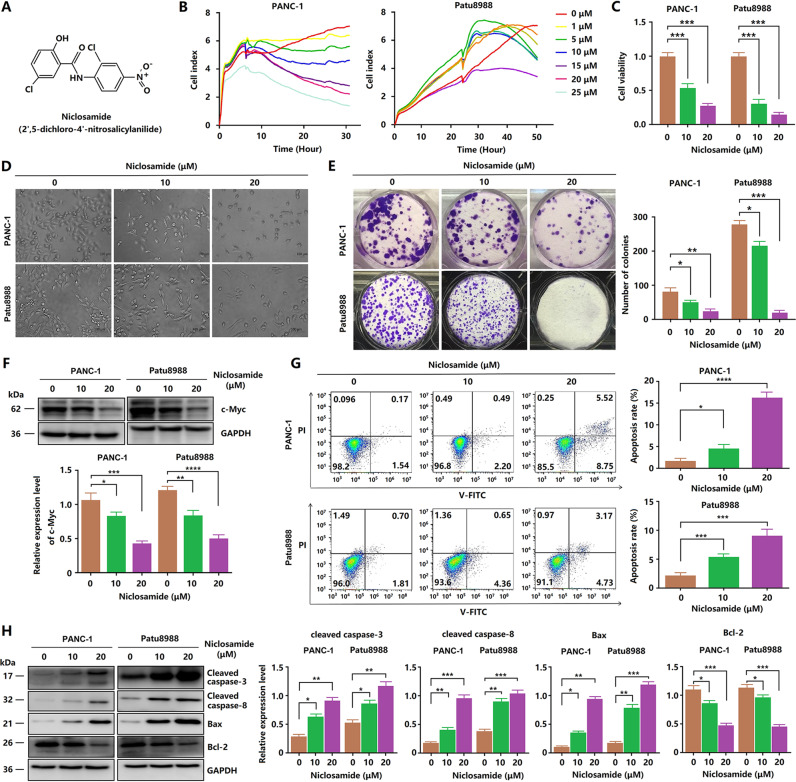
Niclosamide inhibits proliferation and induces apoptosis in PCCs. **A** Molecular structure of niclosamide. **B** Cell proliferation assay was conducted using a xCELLigence RTCA system. **C** Cell viability was assessed by CCK8 assay. **D** Niclosamide affects cell morphology. **E** Clonogenic assays were performed to reveal the effects of niclosamide on the colony formation of PCCs. **G** Apoptosis was determined by flow cytometry. The percentage of total apoptotic cells was quantified for each sample as the sum of early apoptotic and late apoptotic cells. **H** Western blotting analysis of Bcl-2, Bax, cleaved caspase-3, and cleaved caspase-8. **F** Western blotting analysis of c-Myc.

Drug repositioning, which involves the use of existing drugs for different indications, is an effective strategy for traditional drug development. Drug repositioning can decrease treatment costs and development times [14]. The anthelminthic niclosamide (Fig. 1A) has been used to treat tapeworm infection for approximately 50 years [15]. Recent studies have revealed that niclosamide potently inhibits Wnt/β-catenin signaling in various cancers, including oral squamous cell carcinoma, colon cancer, and breast cancer [16–20]. However, the role of niclosamide in PDA remains unknown. Considering the crucial role of the Wnt/β-catenin signaling pathway in pancreatic cancer, we hypothesized that niclosamide could be used to treat patients with PDA. Emerging evidence suggests that activation of the Wnt pathway is linked to tumor progression and resistance to chemotherapy. Moreover, studies have also pointed out that increased Wnt/β-catenin signaling promotes the resistance of PCCs to gemcitabine [21]. Thus, drugs targeting the components of the Wnt pathway may increase the sensitivity of PCCs to gemcitabine [22, 23].

To verify this hypothesis, we evaluated the biological activities of niclosamide in PDA in vitro and in vivo, and further investigated the relevant molecular mechanisms. These data highlight the potential clinical value of niclosamide, shedding a new light on the treatment of gemcitabine-resistant pancreatic cancer.

## Results

### Niclosamide inhibits proliferation and induces apoptosis in PCCs

The effects of niclosamide on Patu8988 and PANC-1 cell proliferation were determined via label-free RTCA, CCK-8 assay, and colony formation assay. After treatment, the antitumor effects of niclosamide on PANC-1 and Patu8988 cells were examined. RTCA showed that the proliferation of PANC-1 and Patu8988 cells decreased significantly after treatment with different niclosamide concentrations (Fig. 1B). Based on the results, two concentrations of niclosamide, 10 and 20 μM, were selected for the following experiments. As shown in Fig. 1C, CCK-8 assay showed that the survival rate of the niclosamide-treated group was much lower than that of the control group (*P* < 0. 05). A significant decrease in the number of tumor cells was also observed under the microscope (Fig. 1D). A plate colony formation assay was also performed to detect the effect of niclosamide on the proliferation of PCCs (Fig. 1E). The colony number of PCCs treated with 10 or 20 μM niclosamide was significantly lower than that of the control PCCs. Western blotting analysis revealed that niclosamide significantly decreased the expression of c-Myc (Fig. 1F). These results suggest that niclosamide inhibits the proliferation of PCCs in a dose-dependent manner.

Two PCC lines were treated with niclosamide for 24 h to examine the effects of niclosamide on cell apoptosis. Apoptotic cells were determined using the Annexin V-FITC/PI method. Compared with that in the control group, the percentage of apoptotic cells in the niclosamide-treated groups increased with increasing niclosamide concentration (Fig. 1G). In particular, after treatment with a high concentration of niclosamide, the degree of apoptosis of PCCs increased considerably. Western blotting analysis also revealed that niclosamide upregulated the expression of cleaved caspase-3, cleaved caspase-8, and Bax, but downregulated the expression of the antiapoptotic protein Bcl-2 (Fig. 1H). These results suggest that niclosamide promotes the apoptosis of PCCs in a dose-dependent manner.

### Niclosamide inhibits the EMT process of PCCs

**Fig. 2:**
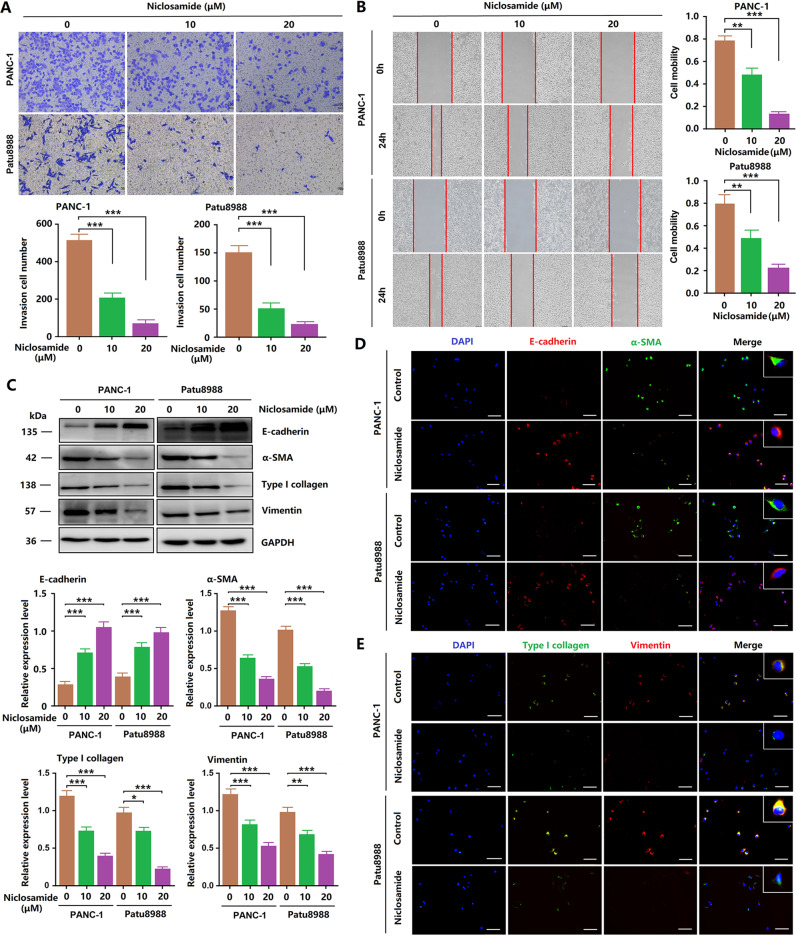
Niclosamide inhibits the EMT process of PCCs. **A** Transwell invasion assay was performed in PCCs. **B** Wound scratch assay, cell mobility = [(the initialized width of the scratch) − (the final width of the scratch)]/(the initialized width of the scratch). **C** Western blotting analysis of E-cadherin, α-SMA, collagen type I, and vimentin (scale bar = 50 μm). **D** Immunofluorescence analysis of E-cadherin in PCCs (scale bar = 50 μm). **E** Immunofluorescence analysis of collagen type I in PCCs (scale bar = 50 μm).

First, PANC-1 and Patu8988 cells were treated with different concentrations of niclosamide for 24 h, and changes in cell morphology were observed under a microscope. Normal PCCs are spindle-like and have more antennae, which are beneficial for migration and invasion. After niclosamide treatment, the cells became round, and the antennae decreased obviously, indicating significant inhibition of the EMT process (Fig. 2A). We performed a Transwell assay to determine the invasive ability of PCCs. As shown in Fig. 2A, the control group showed strong invasive ability, whereas the niclosamide-treated group showed significantly inhibited invasive ability. Next, we performed wound-healing assay to detect the migration of PCCs. Treatment with 10 or 20 μM niclosamide for 24 h markedly reduced the migration ability of PANC-1 and Patu8988 cells (Fig. 2B). Next, the expression of EMT-related essential proteins was detected. As shown in Fig. 2C, niclosamide dramatically increased E-cadherin expression, but decreased α-SMA, collagen type I, and vimentin expression, which further confirmed that niclosamide inhibited the EMT process of PCCs. Immunofluorescence showed the same results (Fig. 2D, E). These findings indicate that niclosamide inhibits EMT in PCCs in a dose-dependent manner.

### Niclosamide decreases Wnt/β-catenin signaling

**Fig. 3:**
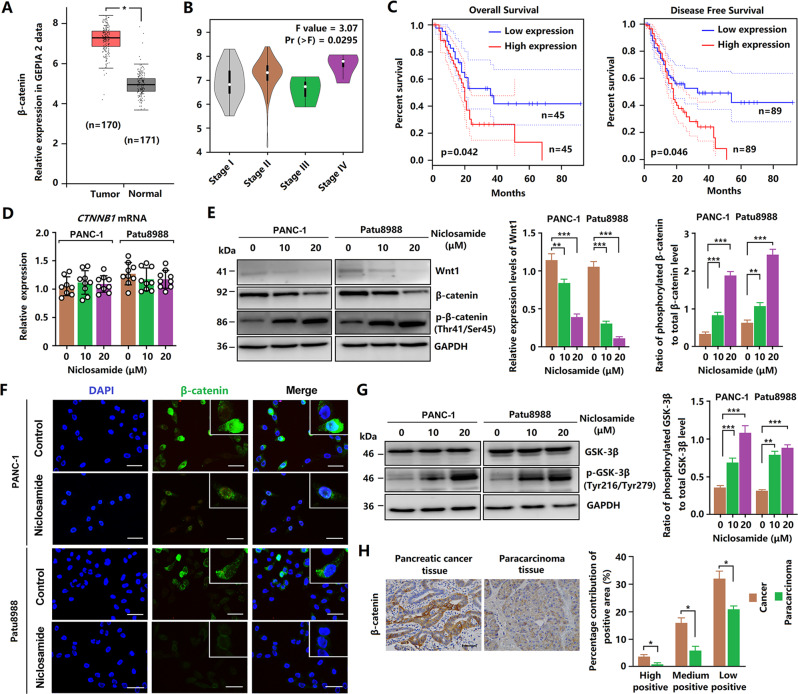
Niclosamide decreases the activity of Wnt/β-catenin signaling. **A** Data of β-catenin expression levels from the GEPIA database. **B** Data of β-catenin gene expression in patients with pancreatic cancer according to the clinical stage, from the GEPIA database. **C** Direct meta-analysis of disease-free survival and overall survival. **D** Relative mRNA expression of β-catenin after niclosamide treatment. **E** Expression of Wnt1, β-catenin, and phosphorylated β-catenin. **F** Immunofluorescence staining of β-catenin protein in PCCs (scale bar = 50 μm). **G** Expression of GSK-3β and phosphorylated GSK-3β. **H** Immunohistochemical staining for β-catenin expression in pancreatic cancer patients (scale bar = 50 μm).

Bioinformatics analysis revealed that β-catenin was highly expressed in PCCs and was related to tumor stage and prognosis (Fig. 3A–C). Therefore, we tested the effect of niclosamide on the β-catenin signaling pathway. We found that niclosamide did not affect the mRNA expression of β-catenin (Fig. 3D). However, western blotting analysis revealed that niclosamide decreased Wnt1 and β-catenin expression, but increased the phosphorylation of β-catenin (Fig. 3E). Considering that β-catenin mainly plays a role in nuclear translocation, we detected the nuclear translocation of β-catenin in PANC-1 and Patu8988 cells via immunofluorescence. As shown in Fig. 3F, niclosamide treatment decreased the nuclear translocation of β-catenin. In addition, niclosamide treatment increased the phosphorylation of GSK-3β (Fig. 3G), which is involved in the degradation of β-catenin. Moreover, the immunohistochemistry analyses showed that β-catenin activity was significantly increased in pancreatic cancer tissues (Fig. 3H). These results suggest that Wnt/β-catenin signaling may be involved in the inhibitory effect of niclosamide in PCCs.

### Niclosamide inhibits tumor xenograft growth in nude mice

**Fig. 4:**
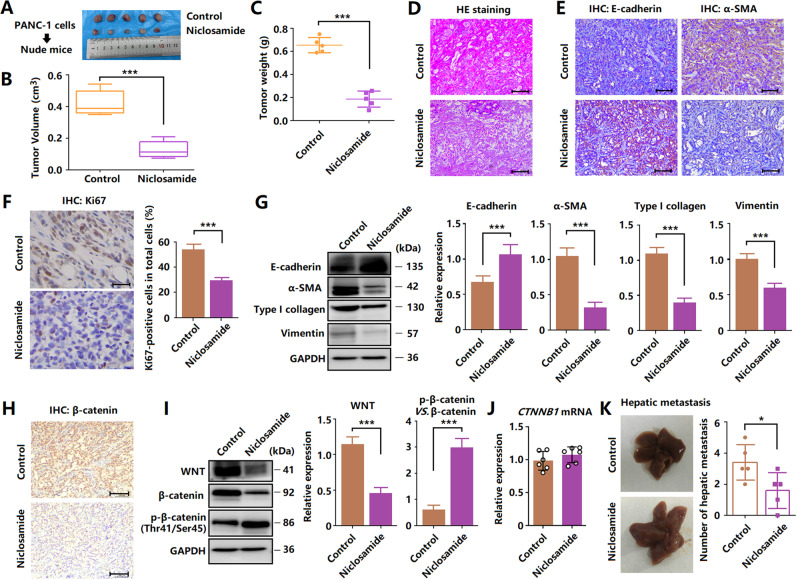
Niclosamide inhibits tumor xenograft growth in nude mice. **A** In vivo mouse xenograft tumor models were established by subcutaneous implantation of PANC-1 cells to nude mice. **B**, **C** The mean tumor volume and weight of tumor xenografts from nude mice over 27 days. **D** Hematoxylin and eosin-stained tumor sections (scale bar = 100 μm). **E** Immunohistochemical staining for E-cadherin and α-SMA (scale bar = 100 μm). **F** Immunohistochemical staining for Ki67 (scale bar = 50 μm). **G** Western blotting analysis of E-cadherin, α-SMA, collagen type I, and vimentin. **J** Relative mRNA expression of β-catenin after niclosamide treatment. **H** Immunohistochemical staining for β-catenin (scale bar = 100 μm). **I** Western blotting analysis of Wnt, β-catenin, and phosphorylated β-catenin. **K** Suppression of hepatic metastasis after niclosamide treatment.

Figure 4A shows a significant difference in tumor volume between the control and niclosamide groups. The average tumor volume and weight were significantly different between the two groups (Fig. 4B, C). The lowest number of tumor cells was observed in the niclosamide group, compared with that in the control group (Fig. 4D). Moreover, western blotting and immunohistochemistry analyses showed that in the niclosamide group, the expression of Ki67, α-SMA, vimentin, and collagen type I was decreased, whereas that of E-cadherin was increased (Fig. 4E–G). The protein expression of Wnt1 and β-catenin significantly decreased with niclosamide treatment, compared with that with DMSO treatment, whereas β-catenin phosphorylation increased (Fig. 4H, I). However, the mRNA level of β-catenin was not different between the control and niclosamide groups (Fig. 4J). In addition, niclosamide treatment reduced tumor metastasis (Fig. 4K). These results are consistent with those obtained in vitro.

### Methyl vanillate and β-catenin overexpression reversed the inhibitory effect of niclosamide in PCCs

**Fig. 5:**
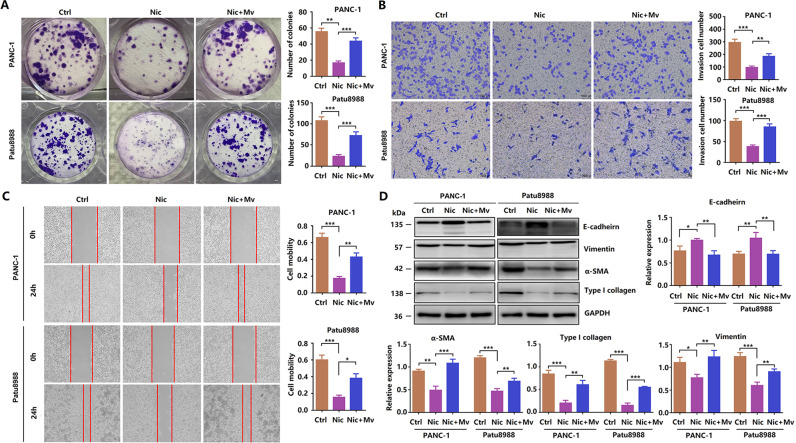
Methyl vanillate and β-catenin overexpression reversed the inhibitory effect of niclosamide in PCCs. **A** In vitro colony-forming assay. **B** Transwell invasion assay was performed in PCCs. **C** Wound scratch assay; cell mobility = [(the initialized width of the scratch) − (the final width of the scratch)]/(the initialized width of the scratch). **D** Western blotting analysis of E-cadherin, α-SMA, collagen type I, and vimentin.

To further verify the inhibitory effect of niclosamide in PCCs via the Wnt/β-catenin signaling pathway, we tested whether its effect using methyl vanillate, a Wnt/β-catenin activator. As shown in Fig. 5A, the numbers of PANC-1 and Patu8988 cell colonies were considerably higher following treatment with 20 μM niclosamide and 20 μM methyl vanillate than after treatment with only 20 μM niclosamide. In addition, Transwell assay results showed that methyl vanillate treatment reversed the inhibitory effect of niclosamide on the invasive ability of PCCs (Fig. 5B). Wound-healing assay showed that methyl vanillate treatment significantly abolished the inhibitory effect of niclosamide on the migration of PANC-1 and Patu8988 cells (Fig. 5C). Next, we detected the expression of EMT-related proteins by western blotting. As shown in Fig. 5D, methyl vanillate reversed the expression of E-cadherin, α-SMA, collagen type I, and vimentin.

**Fig. 6:**
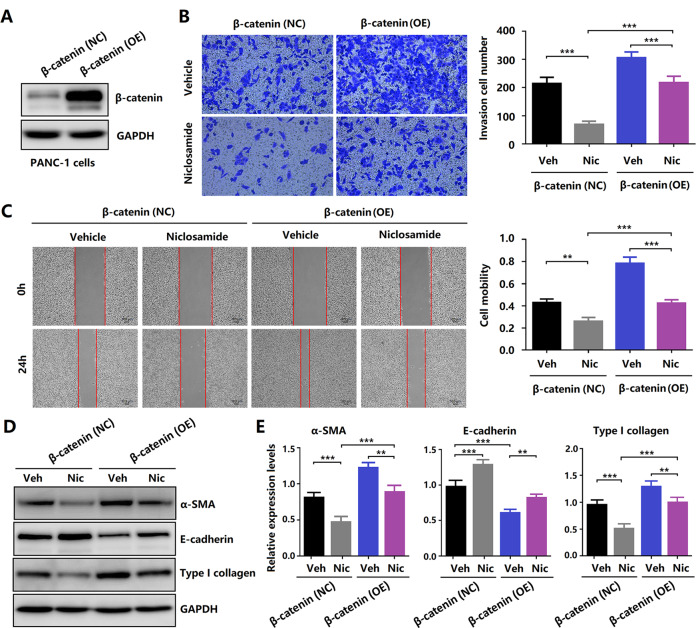
Methyl vanillate and β-catenin overexpression reversed the inhibitory effect of niclosamide in PCCs. **A** Western blotting analyses of protein from the empty vector control (NC) versus β-catenin-OE (OE) cells. **B** Transwell migration assay of PANC-1 cells carrying control (NC) or β-catenin-OE (OE) vectors. **C** Wound scratch assay of PANC-1 cells carrying control (NC) or β-catenin-OE (OE) vectors. **D**, **E** Western blotting analysis of E-cadherin, α-SMA, and collagen type I.

To further study the specific target of niclosamide, β-catenin was overexpressed in PANC-1 cells (Fig. 6A). Moreover, we found that β-catenin overexpression reversed the invasive and migration abilities of niclosamide-treated cells (Fig. 6B, C). The expression of EMT-related proteins was determined by western blotting. Our results revealed that β-catenin overexpression reversed the expression of E-cadherin, α-SMA, and collagen type I (Fig. 6D, E).

### Niclosamide potentiates gemcitabine activity and reduces immune evasion ability in PCCs

**Fig. 7:**
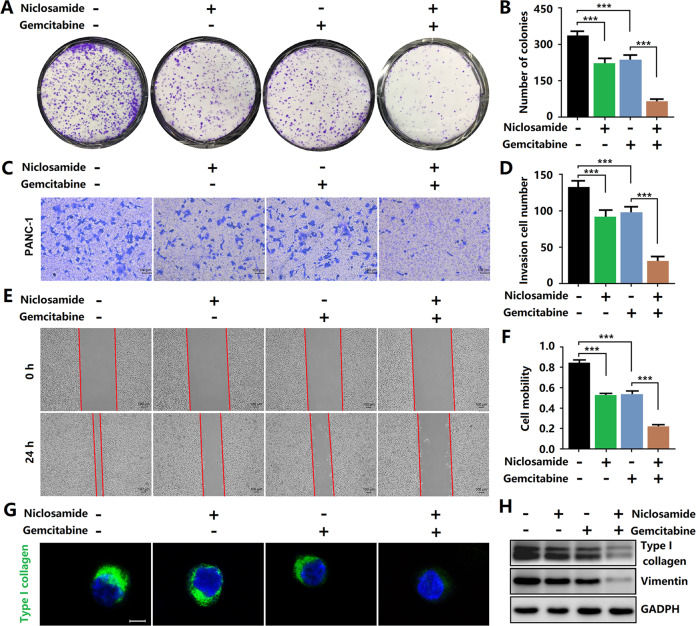
Niclosamide potentiates gemcitabine activity in PCCs. **A**, **B** Clonogenic assays were performed to reveal the effects of niclosamide and/or gemcitabine on the colony formation of PANC-1 cells. **C**, **D** Transwell invasion assay in PANC-1 cells. **E**, **F** Wound scratch assay in PANC-1 cells. **G** Immunofluorescence staining of collagen type I (scale bar = 10 μm) in PANC-1 cells. **H** Western blotting analysis of collagen type I and vimentin.

Gemcitabine is one of the commonly used chemotherapeutics for treating pancreatic cancer, but it is prone to drug resistance, leading to unsatisfactory therapeutic effects. Here, we investigated whether niclosamide enhances the anti-pancreatic cancer effect of gemcitabine. The clone formation experiment results showed that combined treatment with niclosamide enhanced the efficacy of gemcitabine in inhibiting the proliferation of PCCs (Fig. 7A, B). Transwell and wound-healing assays revealed that the invasive and migration ability of PANC-1 cells was significantly reduced by the co-treatment with niclosamide and gemcitabine compared with that after gemcitabine treatment alone (Fig. 7C–F). Simultaneously, immunofluorescence and western blotting assays showed that the combined treatment with niclosamide and gemcitabine significantly reduced collagen type I and vimentin protein expression (Fig. 7G, H).

**Fig. 8:**
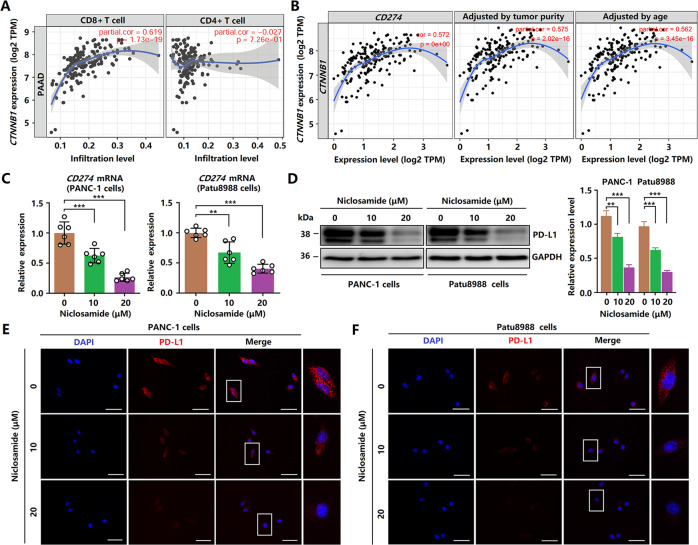
Niclosamide reduces immune evasion ability in PCCs. **A** Correlations between CTNNB1 expression and immune infiltrate density. **B** Correlations between CTNNB1 expression and PD-L1 (CD274) mRNA expression, adjusted by tumor purity and age. **C** PD-L1 (CD274) mRNA expression was examined by quantitative PCR. **D** Western blotting analysis of PD-L1. **E**, **F** Immunofluorescence staining of PD-L1 in PANC-1 and Patu8988 cells (scale bar = 50 μm).

**Fig. 9:**
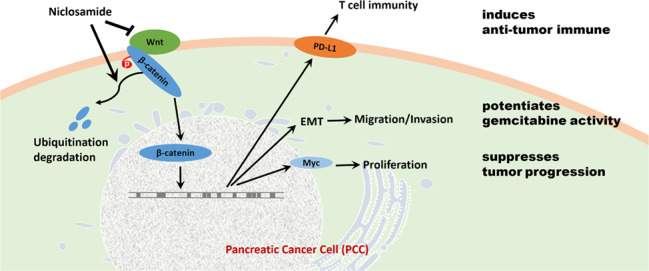
Diagrammatic representation of the role of niclosamide in PCCs. Niclosamide induces GSK-β-mediated β-catenin degradation to potentiate gemcitabine activity, reduce immune evasion ability, and suppress pancreatic cancer progression.

Immune escape plays a vital role in the development and progression of pancreatic cancer. Thus, we also tested the effect of niclosamide on the immune escape of pancreatic cancer. The GEPIA 2 database revealed a clear correlation between ctnnb1 expression and immune infiltration density, as well as between CTNNB1 expression and PD-L1 (CD274) mRNA expression (Fig. 8A, B). As shown in Fig. 8C, qRT-PCR analysis showed that niclosamide inhibited the gene expression of PD-L1 in PCCs. Western blot and immunofluorescence results also showed that niclosamide inhibited PD-L1 in expression in PCCs (Fig. 8D–F), thereby reducing the immune evasion ability of pancreatic cancer (Fig. 9)

## Discussion

Pancreatic cancer is always referred to PDA which is one of the most lethal solid malignancies worldwide. Several first-line chemotherapy agents, such as gemcitabine, have significantly improved the survival of patients many types of cancer. However, these agents have limited outcomes in PDA, and effective drugs for this disease are still lacking [24, 25]. Niclosamide, a known anthelminthic, has been used to treat tapeworm infections [18]. Recent studies have revealed that niclosamide potently inhibits Wnt/β-catenin signaling in various cancers, including oral squamous cell carcinoma, breast cancer, and colon cancer [16–20]. However, studies on the effect of niclosamide in PDA are scarce, and the molecular mechanism of the antitumor activities of niclosamide remains unknown. Thus, in the present study, we investigated the effects of niclosamide on PDA in vitro and in vivo. Our results showed that niclosamide exerts protective effects against cancer by inhibiting the proliferation, migration, and invasion of PANC-1 and Patu8988 PCCs. In addition, we found that a high concentration of niclosamide induced apoptosis of PCCs via the mitochondrial apoptotic pathway, and that niclosamide promoted the activation of caspase-3 and caspase-8 while reducing the expression of the antiapoptotic protein Bcl-2. Further experiment showed that niclosamide treatment inhibited the induction of EMT by Wnt/β-catenin signaling. Furthermore, we established a xenograft nude mouse model to confirm the antitumor bioactivity of niclosamide in vivo. Taken together, our results provided an experimental evidence for the potential of niclosamide as a supplemental treatment to inhibit PDA.

The Wnt/β-catenin signal transduction pathway is a highly conserved pathway in biological evolution, and it plays a crucial role in the development of multiple cancers [26]. In normal somatic cells, β-catenin only acts as a cytoskeleton protein that forms a complex with E-cadherin on the cell membrane to maintain homotypic cell adhesion and prevent cell migration. However, when the extracellular Wnt signaling molecule binds to a specific receptor protein on the cell membrane and activates intracellular disheveled protein, GSK-3β is consequently inactivated, which prevents the phosphorylation and degradation of β-catenin and thus causes the accumulation of β-catenin in the cytoplasm [27]. When β-catenin concentration in the cytoplasm reaches a certain level, it can be transferred to the nucleus. In the nucleus, β-catenin binds to the transcription factor family TCF/LEF, which can activate proto-oncogenes, such as CyclinD1 and c-Myc, which are involved in cell proliferation and differentiation [28]. β-Catenin plays an essential role in this signaling pathway, and its accumulation in the cytoplasm and subsequent translocation to the nucleus is considered a sign that the signaling pathway is activated. Previous studies have confirmed that the Wnt/β-catenin signaling pathway is activated in the development of most human tumors, including PDA [21, 29, 30]. The Wnt/β-catenin signaling pathway mainly modulates cancer cell proliferation and metastasis, and thus regarded as a valuable target of cancer therapy. In our study, niclosamide downregulated the expression of Wnt1, c-Myc, and β-catenin proteins while upregulating that of phosphorylated β-catenin.

For patients with unresectable pancreatic cancer, gemcitabine treatment does not significantly improve their survival rate because of the severity of the primary cancer and acquired resistance to gemcitabine [31]. Therefore, alternative therapeutic options for combination chemotherapy are urgently needed. Several studies have revealed that the activity of the Wnt/β-catenin signaling pathway can be induced and elevated under chemotherapy [32]. Wnt/β-catenin signaling promotes EMT, which is a crucial mechanism of therapeutic resistance that allows tumor cells to escape immune recognition and targeting therapy [33].

Moreover, we detected EMT-related protein expression and found that niclosamide increased E-cadherin expression while decreasing α-SMA, collagen type I, and vimentin expression. Furthermore, we found that methyl vanillate, a Wnt/β-catenin agonist, reversed the inhibitory effect of niclosamide on the EMT process in PDA. These results suggest that the Wnt/β-catenin signaling pathway is inhibited by niclosamide, thereby suppressing the EMT process in PDA [34]. We also examined the effect of niclosamide in combination with gemcitabine and proved that the combined treatment enhanced the efficacy of gemcitabine in inhibiting the proliferation of PCCs. Furthermore, we revealed that niclosamide inhibited PD-L1 expression in PCCs, thereby reducing the cancer-immune evasion ability.

There are still many shortcomings in our study. There are many directions worthy of in-depth discussion. For example, when niclosamide plays an anti-tumor effect, what role does pancreatic stellate cells play? We found that the Wnt/β-catenin signaling pathway plays an important role, but we are not sure whether this pathway plays a dominant role. Furthermore, niclosamide may inhibit the immune escape of PCCs, but this finding remains to be verified by more experiments.

In conclusion, our study showed that niclosamide specifically inhibited Wnt/β-catenin signaling in PDA both in vitro and in vivo (Fig. 9). The inhibitory effect of niclosamide may be mediated via the Wnt/β-catenin signaling pathway. A combined treatment with niclosamide enhanced the efficacy of gemcitabine against PDA, although the clinical effectiveness of the combination of niclosamide and gemcitabine as an antitumor therapy needs further research. Moreover, further studies are needed to explore the bioactive structure of niclosamide.

## Materials and methods

### Drugs

Niclosamide (CAS No: 50-65-7, HPLC: 98.68%) and methyl vanillate (CAS No: 3943-74-6, HPLC: 99.15%) were purchased from MedChemExpress (MCE, NJ, USA).

### Cells and cell culture

Human pancreatic cancer cell lines, Patu8988, and PANC-1, were purchased from the Cell Bank of the Shanghai Institute of Biochemistry and Cell Biology (Shanghai, China). The cells were cultured in Dulbecco’s modified Eagle’s medium (HyClone, Logan, UT, USA) supplemented with 10% fetal bovine serum (FBS; HyClone), 100 µg/ml streptomycin, and 100 U/ml penicillin (Gibco, Billings, MT, USA). The cells were incubated in a humidified 5% CO_2_ incubator at 37 °C.

### CCK-8 assay

The viability of PCCs treated with different concentrations of niclosamide was measured using a Cell Counting Kit 8 (CCK8, HY-K0301; MCE) according to the manufacturer’s instructions. After 24 h of treatment, 10 μl of CCK-8 reagent was added to each well, and which was then incubated for another 2 h. Next, the absorbance at 450 nm was measured, and cell viability was calculated relative to that of untreated control cells.

### Flow cytometry analysis

PANC-1 and Patu8988 cells were cultured with niclosamide for 24 h and then collected by centrifugation (1000 rpm, 10 min, 21 °C). For apoptosis analysis, resuspended cells were incubated with 10 μl Annexin V-FITC and 5 μl propidium iodide (PI) at room temperature (21 °C) for 15–20 min in the dark. Finally, the apoptosis of the PCCs was analyzed by flow cytometry (BD FACSVerse™, BD Biosciences, USA).

### Real-time cellular analysis (RTCA)

For the proliferation assay, the cells were seeded at 4.5 × 10^4^ cells/well in E16-Plate (RTCA, xCELLigence Roche, Penzberg, Germany) for 6 h and then treated with different niclosamide concentrations. The cell growth index was recorded using a label-free real-time cellular analysis (RTCA) system (Roche, Penzberg, Germany).

### Transwell assay

Transwell assays were performed to evaluate the invasive ability of PANC-1 and Patu8988 cells in vitro. Cells at a concentration of 2 × 10^5^ cells in 200 μl of serum-free medium were inoculated in the upper chamber, coated with Matrigel®. Into the lower chamber, 500 μl medium containing 10% FBS was added as a chemoattractant. After incubation for 24 h, the invading cells were fixed with formaldehyde and stained with 0.5% crystal violet (Sigma-Aldrich, Shanghai, China). The number of invading cells was counted in six randomly selected areas under a microscope.

### Colony formation assay

A colony formation assay was performed to evaluate the long-term effect of niclosamide on the clonogenic potential of PCCs. Briefly, 1000–1500 cells were plated per well in six-well plates. Cells were treated with different concentrations of niclosamide once clones were visible to the naked eye. Dimethyl sulfoxide (DMSO) was used as the vehicle control. After 7–10 days of treatment, the cell colonies were stained with 0.5% Crystal Violet and the colonies were counted.

### Wound-healing assay

PCCs were inoculated in six-well plates and incubated at 37 °C for 48 h. A single scratch per plate was made using a small pipette tip creating a 1 mm wide linear gap. The isolated cells were washed away with PBS (phosphate buffered solution), and then medium containing 0, 10, or 20 μM niclosamide was added. The cells were allowed to fill this gap. Images of the culture area were captured using an inverted microscope every 24 h.

### Immunofluorescence staining

Immunofluorescence staining was performed as described previously [35]. PANC-1 and Patu8988 cells were cultured with niclosamide in six-well plates containing glass slides. Cultured cells were washed with PBS and fixed with 4% paraformaldehyde/PBS for 30 min at 4 °C. PCCs were permeabilized with 0.2% Triton/PBS and blocked with 2%BSA/0.2%Triton/PBS. Immunofluorescence staining was performed by incubating the cell sections overnight with the following primary antibodies (Table S1): β-catenin (1:200), E-cadherin (1:200), α-SMA (1: 200), Ptch1 (1: 200), and Smo (1: 200) at 4 °C. After being washed in PBS, the cells were incubated with DyLight 488 (green)- or 594 (red)-labeled secondary antibodies (Sigma-Aldrich) at room temperature for 1 h. Then the nuclei were stained using DAPI (0.5 ug/ml, CST, USA) in PBS for 10 min. The glass slide was dried and then sealed with antifluorescence quenching. Finally, the edges were sealed with clear acrylic nail polish and viewed under fluorescence microscopy.

### Western blotting analysis

Western blotting analysis was performed as described previously [35, 36]. In brief, PANC-1 and Patu8988 cells were seeded in a six-well plate and treated with different concentrations of niclosamide for 24 h. RIPA buffer containing protease and phosphatase inhibitors was used to lyse the cells. After centrifugation (12,000 r.p.m. revolutions per minute) (that is, 13.4 relative centrifugal force, r.c.f.) for 15 min at 4 °C, the supernatants were recovered. Whole proteins were collected from pancreatic cancer cells and tissues, and protein concentrations were determined using a BCA protein assay kit (Beyotime Biotechnology, Shanghai, China). Total protein was denatured and used for western blotting. Primary antibodies were listed in Table S1. The GADPH antibody (1:8,000) was used as the internal reference. The protein bands were visualized using chemiluminescence detection on autoradiographic film.

### Histopathological analysis

The tumor specimens were fixed in formalin, embedded in paraffin, cut into 4 μm sections, and stained with hematoxylin and eosin (Yuanye Biotechnology, Shanghai, China). A DM4000 B LED microscope system (Leica Microsystems, Germany) and a DFC 420C 5 M digital microscope camera (Leica Microsystems) were used to examine the slides and take pictures.

### Nude mouse tumorigenicity

Male nude mice (BALB/c) weighing 18–22 g and 6–8 weeks old (Experimental Animal Center of Wenzhou Medical University, China) were kept in a temperature-humidity light-controlled environment and fed standard food and water. Mice were randomly divided into two groups. PANC-1 cells (5 × 10^6^ cells) in 100 μl of PBS were injected subcutaneously into the right root of experimental mice (*n* = 5), and then the mice were intragastrically administered niclosamide (25 mg/kg·d) daily for 30 days [37, 38]. Model mice (*n* = 5) were injected with 5 × 10^6^ PANC-1 cells and received daily intragastric administration of DMSO (Sigma-Aldrich) as a solvent. Tumors were monitored daily until they became cumbersome or necrotic. The tumor volume was measured every other day based on the formula *V* = (length × width [2])/2, where the length was always the longest dimension of the tumor. The animal study was approved by the Institutional Animal Care and Use Committee of Wenzhou Medical University, China. The animal experiments were performed according to all regulatory and institutional guidelines for animal welfare (National Institutes of Health Publications, NIH Publications No. 80-23) [39].

### Nude mouse liver metastasis tumour models

To establish the liver metastasis model, control or Panc-1 cells (2 × 10^6^) were resuspended in 0.05 mL of phosphate-buffered saline (PBS) and injected into the liver via the tail vein. The mice were sacrificed after 1 month, at which time the livers were harvested.

### Human tissues and evaluation

Pancreatic tumour tissue and their surrounding tissue were obtained by surgical resection from cancer patient. Histologically normal specimens, which were at least 3–5 cm distant from the tumour nodule, were obtained from the corresponding patient. Tissues from patient were collected for studying the expression of β-catenin by immunohistochemical analysis.

### Database analysis

The correlations between the expression of ctnnb1 and the density of the immune infiltrate, as well as between CTNNB1 expression and PD-L1 (CD274) mRNA expression, were evaluated using the GEPIA 2 database (http://gepia2.cancer-pku.cn/#analysis). Data for β-catenin expression levels in PCC patients according to clinical stage, as well as direct meta-analysis for disease-free survival, and overall survival were obtained from the database.

### Statistical analysis

Data are presented as mean ± standard error of the mean. All statistical analyses were performed using the Statistical Package for the Social Sciences (version 16.0; SPSS Inc., Chicago, USA). Student’s t-test was used to compare the mean differences between binary variables. Statistical significance was set at *P* < 0.05.

## Supplementary information

Supplemental Table 1

Reproducibility Checklist
