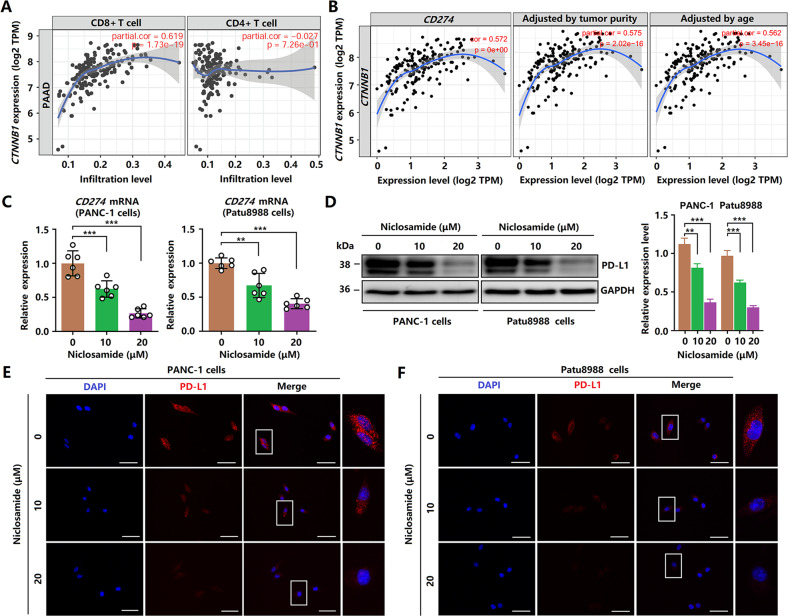# Correction: The anthelmintic drug niclosamide induces GSK-β-mediated β-catenin degradation to potentiate gemcitabine activity, reduce immune evasion ability and suppress pancreatic cancer progression

**DOI:** 10.1038/s41419-022-04705-z

**Published:** 2022-04-19

**Authors:** Yangyang Guo, Hengyue Zhu, Yanyi Xiao, Hangcheng Guo, Miaomiao Lin, Ziwei Yuan, Xuejia Yang, Youze Huang, Qiyu Zhang, Yongheng Bai

**Affiliations:** 1 Key Laboratory of Diagnosis and Treatment of Severe Hepato-Pancreatic Diseases of Zhejiang Province, The First Affiliated Hospital of Wenzhou Medical University, Wenzhou, 325000 China; 2 Department of Laboratory Medicine, The First Affiliated Hospital of Wenzhou Medical University, Wenzhou, 325000 China; 3 Human Genetic Resource Bank, The First Affiliated Hospital of Wenzhou Medical University, Wenzhou, 325000 China; 4 Department of Laboratory Medicine, Wenzhou Hospital of Traditional Chinese Medicine, Wenzhou, 325000 China; 5 Department for Hepatopancreatobiliary Surgery, The First Affiliated Hospital of Wenzhou Medical University, Wenzhou, 325000 China; 6 Center for Health Assessment, Wenzhou Medical University, Wenzhou, 325000 China

**Keywords:** Cancer, Cell death, Pharmacology

Correction to: *Cell Death and Disease* 10.1038/s41419-022-04573-7, published online 03 February 2022.

The original version of this article contained a mistake in figure 8. The correct figure can be found below. The original article has been corrected.