# Supplementary material for: The anthelmintic drug niclosamide induces GSK-β-mediated β-catenin degradation to potentiate gemcitabine activity, reduce immune evasion ability and suppress pancreatic cancer progression
Source: Cell Death Dis. 2022 Feb 3;13(2):112. doi: 10.1038/s41419-022-04573-7 (PMC8814035; doi:10.1038/s41419-022-04573-7)
Supplement: Supplementary file 1 — Supplemental Table 1 [file 41419_2022_4573_MOESM1_ESM.docx]

| **Reagent or Resource** | **Source** | **Identifier** |
| --- | --- | --- |
| Rabbit polyclonal anti-caspase8  Rabbit polyclonal anti-caspase3 | Proteintech  Proteintech | Cat# 13423-1-AP  Cat# 19677-1-AP |
| Rabbit polyclonal anti-Bcl-2 | Proteintech | Cat# 12789-1-AP |
| Rabbit polyclonal anti-Bax | Proteintech | Cat# 50599-2-Ig |
| Rabbit monoclonal anti-α-SMA | ABclonal | Cat# A17910 |
| Rabbit monoclonal anti-E-Cadherin  Rabbit polyclonal anti-Type I collagen | Abcam  Bioworld | Cat# ab231303  Cat# BS70155 |
| Rabbit polyclonal anti-Vimentin | Abcam | Cat# ab45939 |
| Rabbit monoclonal anti-c-Myc  Rabbit polyclonal anti-Wnt1  Rabbit polyclonal anti-β-catenin  Rabbit monoclonal anti-p-β-catenin  Rabbit polyclonal anti-GSK-3β  Rabbit monoclonal anti-p-GSK-3β  Rabbit monoclonal anti-PD-L1 | Abcam  Proteintech  Proteintech  Abcam  Proteintech  Abcam  Abcam | Cat# ab32072  Cat# 27935-1-AP  Cat# 51067-2-AP  Cat# ab81305  Cat# 22104-1-AP  Cat# ab68476  Cat# ab205921 |

**Supplemental Table 1. Primary antibodies in this study**
